# Yearly evolution of organ damage markers in diabetes or metabolic syndrome: data from the LOD-DIABETES study

**DOI:** 10.1186/1475-2840-10-90

**Published:** 2011-10-14

**Authors:** Manuel A Gomez-Marcos, Jose I Recio-Rodríguez, Maria C Patino-Alonso, Cristina Agudo-Conde, Leticia Gomez-Sanchez, Emiliano Rodriguez-Sanchez, Marta Gomez-Sanchez, Luis Garcia-Ortiz

**Affiliations:** 1 Primary Care Research Unit, La Alamedilla Health Centre, Salamanca, Spain; 2 Statistics Department, University of Salamanca, Salamanca, Spain

**Keywords:** Subclinical organ damage, Cardiovascular disease, Type 2 diabetes mellitus, Metabolic syndrome

## Abstract

**Background:**

Cardiovascular disease morbidity-mortality is greater in people with type 2 diabetes mellitus or metabolic syndrome. The purpose of this study was to evaluate the yearly evolution of organ damage markers in diabetes or metabolic syndrome, and to analyze the associated factors.

**Methods:**

An observational prospective study was carried out in the primary care setting, involving 112 patients: 68 diabetics and 44 subjects with metabolic syndrome, subjected to 12 months of follow-up. Measurements: traditional cardiovascular risk factors (blood pressure, blood glucose, lipids, smoking, body mass index (BMI) and) and non-traditional risk factors (waist circumference, hsC Reactive Protein and fibrinogen); subclinical vascular (carotid intima-media thickness, pulse wave velocity and ankle/brachial index), cardiac (Cornell voltage-duration product), renal organ damage (creatinine, glomerular filtration and albumin/creatinine index), and antihypertensive and lipid-lowering drugs.

**Results:**

At baseline, the diabetics presented a mean age of 59.9 years, versus 55.2 years in the subjects with metabolic syndrome (p = 0.03). Diastolic blood pressure, total cholesterol and HDL-cholesterol were lower among the patients with diabetes, while blood glucose and HbA1c, as well as antihypertensive and lipid-lowering drug use, were greater. At evaluation after one year, the diabetics showed a decrease in BMI (-0.39), diastolic blood pressure (-3.59), and an increase in fibrinogen (30.23 mg/dL), ankle/brachial index (0.07) and the number of patients with ankle/brachial index pathologic decreased in 6. In turn, the patients with metabolic syndrome showed an increase in HDL-cholesterol (1-91 mg/dL), fibrinogen (25.54 mg/dL), Cornell voltage-duration product (184.22 mm/ms), ankle/brachial index (0.05) and the use of antihypertensive and lipid-lowering drugs, and a reduction in serum glucose (3.74 mg/dL), HOMA, systolic (-6.76 mmHg), diastolic blood pressure (-3.29 mmHg), and pulse wave velocity (-0.72 m/s). The variable that best predicted a decrease in pulse wave velocity in subjects with metabolic syndrome was seen to be an increase in antihypertensive drug use.

**Conclusions:**

The annual assessment of cardiovascular risk factors and the decrease in pulse wave velocity was more favorable in the patients with metabolic syndrome, probably influenced by the increased percentage of subjects treated with antihypertensive and lipid lowering drugs in this group.

## Introduction

Cardiovascular disease morbidity-mortality is greater in people with type 2 diabetes mellitus (T2DM) or metabolic syndrome [1-4]. The presence of target organ damage (TOD) increases the risk of cardiovascular complications independently of the existing estimated risk [5,6]. In this context, left ventricular hypertrophy (LVH), assessed according to electrocardiographic criteria, increases the risk of coronary complications and stroke [7,8]. The worsening of renal function, assessed by increased creatinine levels, a drop in glomerular filtration rate (GFR), or an increase in protein excretion in urine, increases the risk of cardiovascular diseases [9,10]. Peripheral arterial disease, evaluated by the ankle/brachial index (ABI), is correlated to the development of coronary complications, the incidence of stroke, and cardiovascular mortality [11]. The ultrasound measurement of common carotid artery intima-media thickness (IMTCc) allows the evaluation of vascular structure and the early detection of atherosclerotic lesions, representing a good predictor of future vascular events and a surrogate marker of atherosclerosis [12,13]. IMTCc in T2DM is 0.13 mm greater than in the controls. This implies an age increment of 10 years, and is associated to a 40% increase in cardiovascular risk [14]. Likewise, an increase in arterial stiffness, assessed by pulse wave velocity (PWV), predicts future cardiovascular events and mortality of any cause in both hypertensive subjects and in the general population [15,16], though the role played in individuals with Metabolic syndrome [17,18] or T2DM is not clear [19]. Thus, it is important to know the evolution of the different cardiovascular risk factors and of cardiac, renal and vascular TOD, as well as the corresponding conditioning factors in patients with diabetes or metabolic syndrome.

We postulate that the evolution of the different risk factors and target organ damage is similar in both groups, since the antihypertensive drugs, lipid lowering drugs and metformin with beneficial effects upon blood pressure, lipid profile and target organ damage, are used in greater percentages in the group of patients with T2DM.

The purpose of this study was to evaluate the yearly evolution of organ damage markers in diabetes or metabolic syndrome, and to analyze the associated factors.

## Materials and methods

### Study design and population

A prospective observational study was carried out in a primary care setting. Using consecutive sampling, we included 112 patients with type 2 diabetes mellitus (T2DM) (n = 68) (defined using the American Diabetes Association criteria [20]) or metabolic syndrome (n = 44)(defined according to the National Cholesterol Education Program, ATP III1 definition [21]) from a population of 46,000 people corresponding to two primary care centers (including 2412 diagnosed with diabetes and 4100 with metabolic syndrome). The study included patients with diabetes or metabolic syndrome who visited their family doctor from January 2009 to January 2010, with none of the following exclusion criteria: patients unable to comply with the protocol requirements (psychological and/or cognitive disorders, failure to cooperate, educational limitations and problems for understanding written language, failure to sign the informed consent document); patients participating or programmed to participate in a clinical trial during the study; and patients with serious comorbidities representing a threat to life over the subsequent 12 months. The sample size was estimated to detect as statistically significant a difference in carotid IMT ≥ 0.05 mm between baseline and first year evaluation. Accepting an alpha risk of 0.05 and a beta risk of 0.2 in a two-sided test, and assuming a standard deviation of 0.11 mm, 40 subjects were seen to be necessary, assuming a dropout rate of 5%. The study was approved by an independent ethics committee of Salamanca University Hospital (Spain), and all participants gave written informed consent according to the general recommendations of the Declaration of Helsinki [22]. The LOD-DIABETES Study comprises a cohort of 68 diabetics and 44 subjects with metabolic syndrome subjected to annual evaluation of vascular, renal and cardiac target organ damage (TOD).

### Measurements

A detailed description has been published elsewhere of how the clinical data were collected, the anthropometric measurements were made, blood pressure was recorded, TOD was assessed, and the analytical parameters were obtained [23].

### Blood pressure

Office or clinical blood pressure measurement was obtained by performing three measurements of systolic (SBP) and diastolic blood pressure (DBP), using the average of the last two, with a validated OMRON model M7 sphygmomanometer (Omron Health Care, Kyoto, Japan), and following the recommendations of the European Society of Hypertension [24]. The mean of the last two measurements obtained by the nurse of the research unit from the arm with high blood pressure was used for the study.

### Vascular assessment

#### Assessment of carotid intima-media thickness (IMT)

Carotid ultrasound to assess IMT was performed by two investigators trained for this purpose before starting the study. The reliability of assessment was evaluated before the study, using the intraclass correlation coefficient, which showed values of 0.974 (95%CI: 0.935 to 0.990) for intra-observer agreement on repeated measurements in 20 subjects, and 0.897 (95%CI: 0.740 to 0.959) for inter-observer agreement. According to the Bland-Altman analysis, the limit of inter-observer agreement was 0.022 (95%CI: -0.053 to 0.098), and the limit of intra-observer agreement was 0.012 (95%CI: -0.034 to 0.059). A Sonosite Micromax ultrasound device paired with a 5-10 MHz multifrequency high-resolution linear transducer with Sonocal software was used for performing automatic measurements of IMT in order to optimize reproducibility. Measurements were made of the common carotid after the examination of a longitudinal section of 10 mm at a distance of 1 cm from the bifurcation, performing measurements in the anterior or proximal wall, and in the posterior or distal wall in the lateral, anterior and posterior projections - following an axis perpendicular to the artery to discriminate two lines: one corresponding to the intima-blood interface and the other to the media-adventitious interface. A total of 6 measurements were obtained of the right carotid and another 6 of the left carotid, using average values (average IMT) and maximum values (maximum IMT) calculated automatically by the software. The measurements were obtained with the subject lying down, with the head extended and slightly turned opposite to the exploratory side, following the recommendations of the Manheim Carotid Intima-Media Thickness Consensus [25]. The average IMT was considered abnormal if > 0.90 mm, or if there were atherosclerotic plaques with a diameter 1.5 mm or a focal increase of 0.5 mm or 50% of the adjacent IMT.

#### Evaluation of peripheral artery involvement

This was evaluated using the ankle-brachial index (ABI). The pressure in the extremities was measured using a portable Doppler system Minidop Es-100Vx (Hadeco, Inc. Arima, Miyamae-ku, Kawasaki, Japan) applying the probe at the anterior or posterior tibial artery at an angle of approximately 60° to the direction of blood flow. The ABI was calculated separately for each foot by dividing the greater of the two systolic pressures in the ankle by the greater of the two systolic pressures in the arm. TOD was considered if ABI < 0.9 [24].

#### Pulse wave velocity (PWV)

This parameter was estimated using the SphymgoCor System (AtCor Medical Pty Ltd Head Office, West Ryde, Australia), with the patient in the supine position. The pulse waves of the carotid and femoral arteries were analyzed, estimating the delay with respect to the ECG wave and calculating the corresponding PWV. Distance measurements were taken with a measuring tape from the sternal notch to the carotid and femoral arteries at the sensor location. TOD was considered if PWV > 12 m/s [24].

#### Renal assessment

Kidney damage was assessed by measuring plasma creatinine concentration. Glomerular filtration rate (GFR) was estimated according to the CKD-EPI (Chronic Kidney Disease Epidemiology Collaboration) [26] for Caucasians, and proteinuria was assessed from the albumin/creatinine ratio following the ESH 2007 criteria. TOD was defined as plasma creatinine ≥ 1.3 mg/100 ml in men and ≥ 1.2 mg/100 ml in women; GFR < 60 ml/min; or albumin/creatinine ratio ≥ 22 mg/g in men and ≥ 31 mg/g in women [24].

#### Cardiac assessment

The electrocardiographic examination was performed using a General Electric MAC 3.500 ECG System (General Electric, Niskayuna, NY, USA) that automatically measures the voltage and duration of waves and estimates the criteria of the Cornell voltage-duration product (Cornell VDP) [27]. TOD was defined according to the 2007 European Society of Hypertension/European Society of Cardiology guidelines criteria [24].

Information about kilocalorie intake was collected using the food frequency questionnaire of the University of Navarre, validated for Spain [28]. In order to classify patients as active or non-active cases, we considered sedentary patients as those failing to follow the recommendations of the Centers for Disease Control and Prevention: accumulation of at least 30 min of moderate physical activity 5 or more days a week, or three or more sessions of intense activity a week, each with a minimum duration of 30 min. The individuals performing the different tests were blinded to the clinical data of the patient.

### Statistical analysis

Continuous variables are expressed as the mean ± standard deviation, and qualitative variables as frequency distributions. To analyze the changes in the different variables between the two assessments, use was made of the Student t-test for paired quantitative data, with application of the McNemar test for qualitative variables. The multivariate analysis involved a stepwise multiple linear regression model, using as dependent variables those resulting from the differences in evaluation of the target organ lesions between the two measures (Difference IMT = IMT2-IMT1, Difference PWV = PWV2-PWV1, Difference PDVCORNELL = PDVCORNELL2- PDVCORNELL1, and Difference ABI = ABI2-ABI1). After adjusting for age and sex, we included as independent variables in the analysis for each of the two groups those variables obtained from the differences between the two evaluations that reached statistical significance in the diabetes group (BMI, fibrinogen and DBP) and in the metabolic syndrome group (HDL-cholesterol, baseline blood glucose, fibrinogen, HOMA, SBP, DBP, antihypertensive drugs and lipid-lowering drugs). In a second step using the stepwise method, only those variables which reached statistical significance in the regression model remained in the result of the analysis. The data were analyzed using the SPSS version 18.0 statistical package (SPSS Inc., Chicago, Illinois, USA). A value of p < 0.05 was considered statistically significant.

## Results

Throughout the year of study of follow-up, two males died as a result of acute myocardial infarction, one with T2DM and the other with metabolic syndrome (aged 76 and 65 years, respectively).

At the baseline evaluation, the diabetic patients were 4.5 years older on average (p = 0.03), with a greater percentage of past cardiovascular events, higher C-reactive protein, fibrinogen and HOMA values, and less favorable vascular, renal and cardiac damage indicators, presentando un mayor porcentaje de pacientes activos (45.60% vs 29.50%). In contrast, the subjects with metabolic syndrome showed a poorer lipid profile, and poorer obesity and blood pressure parameters, though for most of the analyzed variables the differences failed to reach statistical significance. Mean antihypertensive and lipid-lowering drug use was greater among the patients with T2DM. La ingesta calórica día fue similar en los dos grupos (2449.29 ± 726.42 vs 2477.08 ± 849.93)

Evaluation after one year, the behavior of the risk factors was similar to that of the data commented above, with a tendency towards improvement of the obesity, lipid profile and blood pressure parameters in both groups. However, IMTCc was seen to equalize in both groups, PWV decreased, and the Cornell voltage-duration product increased in the patients with metabolic syndrome. Antihypertensive and lipid-lowering drug use increased in the subjects with metabolic syndrome (Tables 1, 2, 3 and 4).

**Table 1:** General demographic and clinics characteristics in Diabetics

Variables	Basal evaluation	Annual review	Differences	IC 95%		p Value	
Number (%)	68 (60.70)	67 (58.90)					
Age (years)	59.91 ± 10.08	60.91 ± 10.08					
Males n (%)	43 (63.20)	42 (62.68)					
Years of evolution	5.41 ± 4.19	6.41 ± 4.19					
Smokers n (%)	16 (23.50)	16(25.00)					
Ischemic heart disease n (%)	8 (11.80)	8 (12.50)					
Cerebrovascular disease n (%)	2 (2.90)	2(2.50)					
BMI (kg/m^2^)	30.08 ± 4.96	29.71 ± 5.28	-0.39	-0.69	to	-0.08	0.014
Waist circumference (cm)	102.93 ± 12.73	101.63 ± 13.74	-1.30	-2.08	to	0.11	0.078
Total Cholesterol (mg/dL)	187.54 ± 33.97	185.84 ± 37.10	-1.70	-10.36	to	5.43	0.534
Tryglicerides (mg/dL)	143.90 ± 68.25	141.63 ± 76.03	-2.24	-14.90	to	13.62	0.929
LDL cholesterol (mg/dL)	108.61 ± 28.45	107.95 ± 28.91	-0.66	-8.33	to	5.28	0.656
HDL cholesterol (mg/dL)	48.61 ± 11.69	48.48 ± 12.09	-0.13	-2.06	to	1.52	0.764
Serum glucose (mg/dL)	126.68 ± 35.34	132.48 ± 45.81	5.80	-0.45	to	14.54	0.065
HbA1c (%)	6.83 ± 1.17	7.01 ± 1.33	0.18	-0.07	to	0.41	0.159
Serum creatinine, (mg/dL)	0.86 ± 0.17	0.86 ± 0.21	0.00	-0.04	to	0.04	1.000
hs-c-reactive (mg/dL)	0.34 ± 0.51	0.32 ± 0.42	-0.02	-0.17	to	0.10	0.631
Fibrinogen (mg/dL)	337.16 ± 61.16	365.82 ± 93.23	26.66	6.18	to	54.28	0.015
HOMA-IR	3.24 ± 2.69	3.34 ± 3.82	1.00	-0.38	to	1.24	0.287
Office SBP (mm Hg)	136.13 ± 19.09	132.44 ± 18.80	-3.69	-8.16	to	1.16	0.139
Office DBP (mm Hg)	82.64 ± 11.59	78.92 ± 9.82	-3.72	-6.08	to	-1.10	0.005
Office PP, mm Hg	53.88 ± 14.38	54.69 ± 17.64	0.81	-2.65	to	4.27	0.640
Office HR	72.10 ± 12.43	70.56 ± 11.06	-1.54	-2.81	to	1.36	0.488
Mean Antihypertensive Drugs	1.51 ± 1.15	1.57 ± 1.21	0.06	-0.17	to	0.28	0.603
Antihypertensive Drugs, n (%)	52 (76.50)	51(75.00)	-1.5	-1.4	to	1.5	0.625
Mean Lipid lowering drugs	0.68 ± 0.56	0.66 ± 0.56	-0.01	-0.10	to	0.07	0.742
Lipid lowering drugs, n (%)	45 (66.20)	44(64.70)	-1.5	-1.8	to	1.6	0.687

Data for qualitative variables are expressed as N: number (%) and quantitative variables as mean ± standard deviation.

BMI: body mass index; LDL: low density lipoprotein; HDL: high density lipoprotein; HbA1C: glycosylated hemoglobin; HOMA-IR: homeostasis model assessment insulin resistance; SBP: Systolic blood pressure; DBP: Diastolic blood pressure; PP: pulse pressure; HR: heart rate.

**Table 2:** General demographic and clinics characteristics in Metabolic Syndrome

Variables	Basal evaluation	Annual review	Differences	IC 95%		p Value	
Number (%)	44	43					
Age (years)	55.20 ± 12.49	56.20 ± 12.49					
Males n (%)	28 (63.60)	27 (62.79)					
Years of evolution	1.58 ± 2.06	1.58 ± 2.06					
Smokers n (%)	9 (20.50)	9 (20.93)					
Ischemic heart disease n (%)	2 (4.50)	2 (4.65)					
Cerebrovascular disease n (%)	0 (0.00)	0 (0.00)					
BMI (kg/m^2^)	31.08 ± 3.52	30.82 ± 4.05	-0.26	-0.61	to	0.05	0.094
Waist circumference (cm)	104.75 ± 9.76	104.79 ± 8.98	0.04	-1.34	to	1.52	0.896
Total Cholesterol (mg/dL)	218.95 ± 44.15	208.51 ± 41.26	-10.44	-21.60	to	0.06	0.051
Tryglicerides (mg/dL)	167.68 ± 53.07	151.02 ± 83.70	-16.66	-38.47	to	7.54	0.182
LDL cholesterol (mg/dL)	140.25 ± 39.67	130.56 ± 35.94	-9.69	-19.85	to	0.04	0.051
HDL cholesterol (mg/dL)	45.18 ± 10.96	47.38 ± 11.42	2.20	0.16	to	3.65	0.033
Serum glucose (mg/dL)	92.57 ± 11.78	88.91 ± 12.52	-3.66	-7.02	to	-0.47	0.026
HbA1c	5.59 ± 0.66	5.67 ± 0.32	0.08	-0.11	to	0.30	0.367
Serum creatinine, (mg/dL)	0.89 ± 0.16	0.90 ± 0.19	0.01	-0.03	to	0.05	0.575
hs-c-reactive (mg/dL)	0.26 ± 0.21	0.27 ± 0.25	0.01	-0.04	to	0.05	0.687
Fibrinogen (mg/dL)	327.05 ± 59.07	345.16 ± 66.64	18.11	6.87	to	44.21	0.009
HOMA-IR	2.96 ± 1.82	2.22 ± 1.33	-0.74	-1.35	to	-0.27	0.004
Office SBP (mm Hg)	142.43 ± 12.46	134.95 ± 15.52	-7.48	-11.35	to	-2.18	0.005
Office DBP (mm Hg)	88.63 ± 9.61	84.92 ± 10.26	-3.71	-6.32	to	-0.26	0.034
Office PP, mm Hg	54.23 ± 11.35	50.77 ± 12.78	-3.46	-6.96	to	0.73	0.109
Office HR	74.23 ± 12.23	72.44 ± 11.66	-1.79	-3.78	to	0.94	0.232
Mean Antihypertensive Drugs	0.82 ± 1.04	1.34 ± 1.20	0.52	0.21	to	0.83	0.002
Antihypertensive Drugs, n (%)	21 (47.70)	31 (72.10)	24.40	-44	to	40	0.001
Mean Lipid lowering drugs	0.30 ± 0.46	0.43 ± 0.55	0.13	0.03	to	0.24	0.013
Lipid lowering drugs, n (%)	13 (29.50)	19 (44.20)	14.70	-3.5	to	450.0	0.031

Data for qualitative variables are expressed as N: number (%) and quantitative variables as mean ± standard deviation.

BMI: body mass index; LDL: low density lipoprotein; HDL: high density lipoprotein; HbA1C: glycosylated hemoglobin; HOMA-IR: homeostasis model assessment insulin resistance; SBP: Systolic blood pressure; DBP: Diastolic blood pressure; PP: pulse pressure; HR: heart rate.

**Table 3:** Target organ damage in Diabetics

Variables	Basal evaluation	Annual review	Differences	IC 95%		p Value	
**Vascular**							

Carotid IMT men average (mm)	0.76 ± 0.12	0.76 ± 0.10	0.00	-1	to	1	0.82
Carotid IMT men average ≥ 90 mm, n (%)	17 (25.00)	15 (22.10)	-2.90	-4.7	to	1.2	0.63
Carotid IMT maximum average (mm)	0.94 ± 0.14	0.94 ± 0.13	0.00	-0.01	to	0.01	0.74
Carotid IMT maximum average ≥ 90 mm, n (%)	46 (67.60)	42 (61.80)	-5.80	-11	to	21	0.39
Plaques, n (%)	15 (22.10)	15 (22.10)	10	-14	to	14	1.00
ABI	1.10 ± 0.13	1.17 ± 0.08	0.07	0.04	to	0.11	< 0.001
TOD ABI, n (%)	8 (11.80)	2 (3.10)	-8.70	-17	to	10	0.03
PWV, (m/s)	9.59 ± 2.32	9.78 ± 2.49	0.19	-0.26	to	0.77	0.33
PWV ≥ 12 m/s, n (%)	10 (15.20)	11 (17.50)	2	-10	to	14	0.63
TOD Vascular, n (%)	25 (37.90)	23 (36.50)	-14	-18	to	-10	0.75

**Renal**							

GFR CKD-EPI (mL/min/1.73 m2)	87.95 ± 13.06	88.64 ± 15.35	0.69	-2.09	to	3.32	0.65
TOD GFR CKD-EPI ≤ 60 mL/min/1.73 m2, n(/%)	1 (1.50)	3 (4.70)	3	-3	to	15	0.50
Albumin/creatinine (mg/g)	36.09 ± 82.81	34.88 ± 75.98	-1.21	-20.80	to	15.05	0.75
TOD Albumin/creatinine (mg/g), n (%)	11 (16.20)	9 (14.50)	-1.70	-15	to	9	0.06
TOD Renal n (%)	13 (20.00)	12 (19.40)	-0.60	-0.74	to	-0.50	0.63

**Heart**							

Cornell VDP (mmms)	1648.29 ± 654.68	1584.79 ± 522.69	-29.91	-118.71	to	58.88	0.50
TOD Cornell VDP (mmms) patologico, n(%)	7 (10.30)	5 (7.80)	-2.50	-12	to	7	0.25
TOD global, n (%)	34 (51.50)	32 (50.80)	-0.7	-1.9	to	1.5	0.75

Data for qualitative variables are expressed as N° (%) and quantitative variables as mean ± standard deviation.

IMT: Intima-media thickness; ABI: ankle-brachial index; TOD: Target organ damage: PWV: Pulse wave velocity; GFR: glomerular filtration rate; CKD-EPI: Chronic Kidney Disease Epidemiology Collaboration; PDV: Voltage-duration product

**Table 4:** Target Organ damage in Metabolic Syndrome

Variables	Basal evaluation	Annual review	Differences	IC 95%		p Value	
**Vascular**							

Carotid IMT men average (mm)	0.75 ± 0.12	0.76 ± 0.12	0.00	-0.01	to	0.02	0.40
Carotid IMT men average ≥ 90 mm, n (%)	6 (13.60)	5 (11.40)	-2.20	-16	to	11	0.62
Carotid IMT maximum average (mm)	0.93 ± 0.15	0.93 ± 0.15	0.00	-0.02	to	0.03	0.63
Carotid IMT maximum average ≥ 90 mm, n (%)	25 (56.80)	23 (52.30)	-4.50	-24	to	18	0.39
Plaques, n (%)	3 (6.80)	3 (6.90)	0.1	-10	to	11	1.00
ABI	1.10 ± 0.10	1.15 ± 0.09	0.05	0.01	to	0.09	0.03
TOD ABI, n (%)	1(2.30)	2 (4.70)	2.40	-5	to	10	0.03
PWV, (m/s)	9.34 ± 2.69	8.57 ± 2.22	-0.77	-1.50	to	0.07	0.04
PWV ≥ 12 m/s, n (%)	7 (15.90)	2 (4.70)	-11.20	-24	to	12	0.75
TOD Vascular, n (%)	11 (25.00)	6 (14.00)	-11	-27	to	54	0.06

**Renal**							

GFR CKD-EPI (mL/min/1.73 m2)	88.57 ± 13.89	87.26 ± 16.03	-1.31	-5.15	to	2.58	0.50
TOD GFR CKD-EPI ≤ 60 mL/min/1.73 m2, n(/%)	0 (0.00)	3 (7.00)	7	-1	to	15	0.25
Albumin/creatinine (mg/g)	17.31 ± 39.04	28.89 ± 133.82	11.58	-30.36	to	55.92	0.55
TOD Albumin/creatinine (mg/g), n (%)	7 (16.30)	1 (2.40)	13.90	-25	to	-2	0.06
TOD Renal n (%)	7 (16.30)	5 (11.90)	-4.40	-18	to	10	1.00

**Heart**							

Cornell VDP (mmms)	1486.64 ± 457.67	1661.89 ± 536.983	184.22	62.28	to	306.15	0.01
TOD Cornell VDP (mmms) patologico, n(%)	1 (2.30)	4 (9.30)	7	-3	to	17	0.25
TOD global, n (%)	1 (2.30)	4 (9.30)	7	-3	to	17	0.25

Data for qualitative variables are expressed as N° (%) and quantitative variables as mean ± standard deviation.

IMT: Intima-media thickness; ABI: ankle-brachial index; TOD: Target organ damage: PWV: Pulse wave velocity; GFR: glomerular filtration rate; CKD-EPI: Chronic Kidney Disease Epidemiology Collaboration; PDV: Voltage-duration product

**Figure 1:**
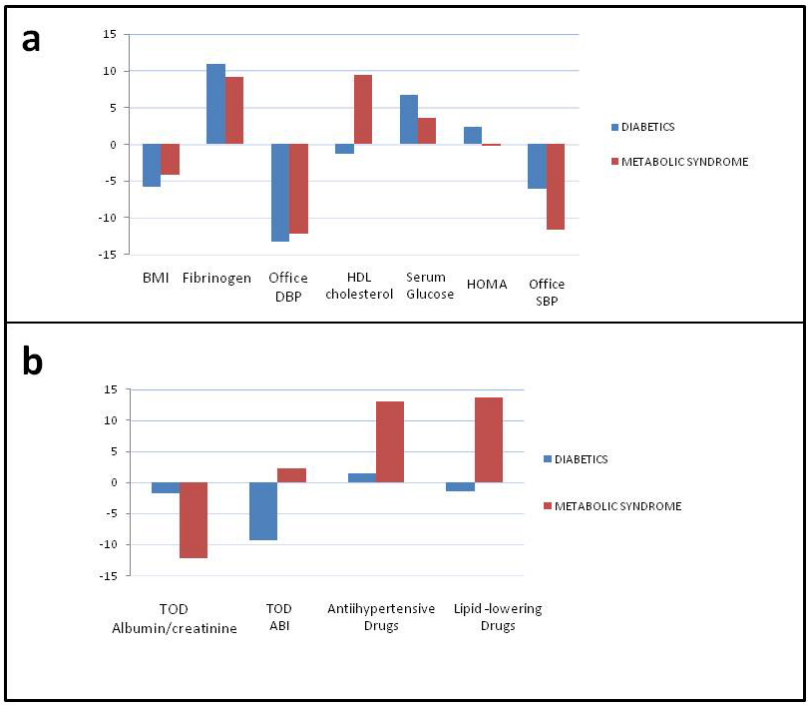
**Changes between baseline and evaluation after one year of follow-up**. Changes between baseline and evaluation after one year of follow-up in target organ damage (a) and other study variables (b) in subjects with T2DM or Metabolic syndrome, adjusting the values of the differences to a scale of -100 to 100. IMT: intima-media thickness; ABI: ankle/brachial index; PWV: pulse wave velocity; VDP: voltage-duration product; BMI: body mass index; SBP: systolic blood pressure; DBP: diastolic blood pressure.

On analyzing the differences in the risk factors, vascular, renal and cardiac TOD and antihypertensive and lipid-lowering drugs between baseline and the evaluation after one year, a significant decrease was noted in BMI and diastolic blood pressure, together with an increase in fibrinogen and ABI in the patients with T2DM. In contrast, the patients with an initial diagnosis of Metabolic syndrome showed a significant drop in systolic and diastolic blood pressure, HOMA index and PWV, and an increase in HDL-Cholesterol, serum glucose, fibrinogen, ABI, Cornell voltage-duration product and antihypertensive and lipid-lowering drug use (Tables 1, 2, 3 and 4, Figure 1).

In the multiple regression analysis, the variables that remained in the equation explaining the changes in TOD parameters in patients with Metabolic syndrome were: the difference in antihypertensive drug use in PWV (R^2 ^= 0.235), and the difference in antihypertensive and lipid-lowering drug use in relation to the Cornell voltage-duration product (R^2 ^= 0.258). However, in the diabetic patients and following the same regression analysis, the variable remaining in the model explaining the differences in PWV (R^2 ^= 0.133) and Cornell voltage-duration product (R^2 ^= 0.104) was seen to be the difference in systolic blood pressure (Table 5).

**Table 5:** Factors influencing the differences between the two evaluations of target organ damage in diabetics and patients with metabolic syndrome, in the multiple regression analysis

Diabetics	Metabolic syndrome						
	***β***	**IC 95%**	**p**		***β***	**IC 95%**	**p Value**

D-IMT Adjusted R2 = 0.001		D-IMT Adjusted R2 = 0.038					

Constant	-0.01	-0.09 to 0.09	0.97	constant	0.04	0.06 to 0.14	0.40
Age	-0.01	-0.01 to 0.01	0.43	Age	-0.01	0.01 to 0.01	0.14
Gender	0.02	-0.01 to 0.05	0.21	Gender	0.02	0.02 to 0.06	0.32

**D-PWV Adjusted R^2 ^= 0.133**		**D-PWV Adjusted R^2 ^= 0.235 **					

Constant	-1.97	-6.87 to 2.93	0.42	constant	2.57	1.34 to 6.48	0.19
Age	0.04	-0.04 to 0.12	0.28	Age	-0.05	0.12 to 0.01	0.09
Gender	0.22	-1.07 to 1.51	0.73	Gender	1.13	0.59 to 2.86	0.19
D-DBP	0.05	0.01 to 0.08	0.01	D-FHTA	-1.44	2.39 to -0.50	0.01

**D-PDV DE CORNELL Adjusted R^2 ^= 0.104**		**D-PDV DE CORNELL Adjusted R^2 ^= 0.258**					

Constant	124.16	-889.48 to 1137.79	0.80	constant	-74.32	-600.14 to 451.49	0.77
Age	-2.57	-18.74 to 13.59	0.75	Age	4.63	4.15 to 13.421	0.29
Gender	-18.44	-248.79 to 285.66	0.89	Gender	-19.19	251.72 to 213.33	0.87
D-DBP	7.89	0.88 to 14.90	0.03	D-FHTA	-440.80	139.92 to 741.69	0.01

**D-ABI Adjusted R^2 ^= 0.036**		**D-ABI Adjusted R^2 ^= 0.005**					

Constant	-0.095	-0.25 to 0.06	0.22	constant	-0.06	0.25 to 0.12	0.51
Age	0.02	0.01 to 0.04	0.12	Age	0.01	0.01 to 0.01	0.36
Gender	0.04	-0.01 to 0.10	0.14	Gender	0.01	0.03 to 0.03	0.23

Dependent variable: D-IMT: difference Intima-media thickness; D-PWV: difference pulse wave velocity; D-PDV DE CORNELL: difference voltage-duration product CORNELL. D-ABI: difference ankle-brachial index

Adjustment variables:Age; Gender: (male = 1; female = 0).

Independent variables: D-BMI: difference body mass index; D-Fibrinogen: difference fibrinogen; D-HDL: difference high density lipoprotein; D-serum glucose: difference serum glucose; D-HOMA-IR: difference homeostasis model assessment insulin resistance; D-SBP: difference sistolic blood pressure; D-DBP: difference diastolic blood pressure. D-FHTA: D-Antihypertensive Drugs; D-FHPL: D-Hypolipidemic drugs.

R2: determination coefficient; p: statistically significant differences (p < 0.05).

## Discussion

The data obtained describe the evolution of to conventional and non-conventional risk factors and of las TOD vascular, cardiac and renal damage in patients with T2DM or metabolic syndrome over one year of follow-up. We observed a increase in ABI in the diabetics. In contrast, the patients with metabolic syndrome experienced an increase in ABI and Cornell voltage-duration product, and a decrease in PWV, probably as a result of an increased use of antihypertensive drugs.

The mean IMTCc in the patients with T2DM was 0.76 mm, same in the two assessments. This being similar (taking age into account) to the data obtained in Caucasians with T2DM in the metaanalysis published by Brohall et al. [14]. The mean IMTCc in the two evaluations among the subjects with Metabolic syndrome was 0.75 mm and greater than the data of the Carmela study (0.69 mm). These differences are probably explained by patient age, since in this study the subjects with Metabolic syndrome were comparatively younger [29].

The mean PWV values in the diabetics (9.59 m/sec at initial measurement and 9.78 m/sec after one year of follow-up) were similar to those reported by Lacy et al. [30], and higher than those recorded in population-based studies [16,31]. In the patients with metabolic syndrome, the mean PWV decreased from 9.34 to 8.57 m/sec, a situation explained by the increased use of antihypertensive treatment (from 0.82 to 1.34 drugs per patient), which is presently the most potent option for reducing arterial stiffness [32]. Likewise, antihypertensive drug use is the variable explaining the variability in the differences in the regression analysis.

The behavior of ABI was similar in both patient groups, with an increase after one year of follow-up. Similar data have been published by Ito et al. [33] in patients with T2DM. However, it must be remembered that in diabetic patients the standard threshold sensitivity (0.9) is lower as a result of which the efficiency of ABI is limited. Moreover, in this group of patients the sensitivity for values between 0.9-1.3 is low (15-79%) [34,35]. In the improved results of ABI, one of the possible causes for the increase in the first year is the decrease in brachial systolic pressure, without an accompanying decrease at pedal or tibial artery due to the atherosclerosis of these patients, in addition to the between- and within-observer variability there may be between the two measurements.

The evaluation of left ventricular hypertrophy based on the Cornell voltage-duration product did not vary between the two evaluation time points in the diabetic patients, though it was seen to increase at the second measurement in the patients with metabolic syndrome. Apart from the low sensitivity (31%) of electrocardiography in detecting left ventricular hypertrophy, the electrocardiographic criteria are of little diagnostic use in the isolated interpretation of a patient with left ventricular hypertrophy [8]. In any case, the ELECTROPRES platform, implemented in several Spanish centers, has concluded that the criteria of the Lewis index (R-I+ S-III) and Cornell product ([R-aVL + S-V3] [+ 6 in women]) [36]were those which detected most cases of left ventricular hypertrophy.

There were no differences in glomerular filtration rate estimated with the CKD-EPI equation either between evaluation timepoints or between groups the values in all cases being over 85 ml/min/1.73 m^2 ^and greater than those recorded in other studies [33,37]. Likewise, we observed no differences between the two evaluations in terms of the albumin-creatine index the values being lower in the subjects with Metabolic syndrome, and also lower than the values published for diabetic subjects in our setting [37].

This study has some limitations that must be considered when interpreting the results obtained. Firstly, the number of subjects per group, which limits the power of an analyses made, as well as follow-up limited to the first year of the study. It also should be taken into account that selection was not randomized but involved consecutive sampling, and the two groups are not fully balanced in terms of age (4 years of difference) - a fact that may influence the course, though the analyses have been adjusted for this variable in order to minimize its influence.

The annual assessment of cardiovascular risk factors and the decrease in pulse wave velocity was more favorable in the patients with metabolic syndrome, probably influenced by the increased percentage of subjects treated with antihypertensive and lipid lowering drugs in this group.

## Abbreviations

T2DM: Diabetes mellitus tipo 2; TOD: target organ damage; LVH: Left ventricular hypertrophy; IMT: Intima-media thickness; PWV: Pulse wave velocity; IMTCc: Intima-media thickness of the common carotid artery; ABI: Ankle-brachial index; PWV: Pulse wave velocity; SBP:Systolic blood pressure; DBP: Dyastolic blood pressure; PP: Pulse pressure; GFR: glomerular filtration rate; CKD-EPI: Chronic Kidney Disease Epidemiology Collaboration.

## Competing interests

The authors declare that they have no competing interests.

## Authors' contributions

MAGM devised the study, designed the protocol, participated in fund raising, interpretation of results, prepared the manuscript draft and corrected the final version of the manuscript. JIRR and CAC participated in the study design, data collection and manuscript review. MCPA performed all analytical methods, interpretation of results, and manuscript review. ERS, LGS and LGS participated in the study design, interpretation of results, and manuscript review. LGO participated in the protocol design, fund raising, analysis of results, and final review of the manuscript. Finally, all authors reviewed and approved the final version of the manuscript.
